# MSC from fetal and adult lungs possess lung-specific properties compared to bone marrow-derived MSC

**DOI:** 10.1038/srep29160

**Published:** 2016-07-06

**Authors:** Sara Rolandsson Enes, Annika Andersson Sjöland, Ingrid Skog, Lennart Hansson, Hillevi Larsson, Katarina Le Blanc, Leif Eriksson, Leif Bjermer, Stefan Scheding, Gunilla Westergren-Thorsson

**Affiliations:** 1 Department of Experimental Medical Science, Lung Biology Unit, Lund University, Lund, Sweden; 2 Department of Respiratory Medicine and Allergology, Lund University and Skåne University Hospital, Lund, Sweden; 3 Division of Clinical Immunology; Centre for Allogeneic Stem Cell Transplantation, Karolinska Institutet, Huddinge University Hospital, Stockholm, Sweden; 4 Lund Stem Cell Center, Lund University, Lund, Sweden; 5 Department of Hematology, Skåne University Hospital, Lund, Sweden

## Abstract

Mesenchymal stromal cells (MSC) are multipotent cells with regenerative and immune-modulatory properties. Therefore, MSC have been proposed as a potential cell-therapy for bronchiolitis obliterans syndrome (BOS). On the other hand, there are publications demonstrating that MSC might be involved in the development of BOS. Despite limited knowledge regarding the functional role of tissue-resident lung-MSC, several clinical trials have been performed using MSC, particularly bone marrow (BM)-derived MSC, for various lung diseases. We aimed to compare lung-MSC with the well-characterized BM-MSC. Furthermore, MSC isolated from lung-transplanted patients with BOS were compared to patients without BOS. Our study show that lung-MSCs are smaller, possess a higher colony-forming capacity and have a different cytokine profile compared to BM-MSC. Utilizing gene expression profiling, 89 genes including lung-specific FOXF1 and HOXB5 were found to be significantly different between BM-MSC and lung-MSC. No significant differences in cytokine secretion or gene expression were found between MSC isolated from BOS patients compared recipients without BOS. These data demonstrate that lung-resident MSC possess lung-specific properties. Furthermore, these results show that MSC isolated from lung-transplanted patients with BOS do not have an altered phenotype compared to MSC isolated from good outcome recipients.

Mesenchymal stromal cells (MSC) have potent immune-regulatory and regenerative functions and they are therefore promising candidates for cell therapy approaches to treat a variety of different diseases including severe diseases of the lung, such as idiopathic pulmonary fibrosis (IPF)^1^, chronic obstructive pulmonary disease (COPD)^2^ and acute respiratory distress syndrome (ARDS)^3^. The only curative treatment of severe lung diseases like IPF and COPD at present is lung-transplantation. However, chronic rejection, which is manifested as bronchiolitis obliterans syndrome (BOS)/obliterative bronchiolitis, is a severe complication affecting the survival after a lung-transplantation. The hallmark of this complication is the fibrotic obliteration of the peripheral airways^4^, which is a fibro-proliferative disease for which inflammation has been shown to be an important driving factor. It has therefore been suggested that MSC might be a treatment option.

Although MSC have been used in clinical trials for the treatment of severe lung diseases, not much is known about the primary resident lung-MSC. Open questions are for example if the lung-MSC are altered in diseases such as BOS and how lung-resident MSC differ from the bone marrow (BM)-derived MSC, the predominant MSC source in clinical trials? Therefore, the current study aimed to investigate the tissue specificity of MSC isolated from lung tissues (fetal and adult) and to compare them to the extensively studied BM-derived MSC. Furthermore, we aimed to investigate whether MSC isolated from lung-transplanted patients with BOS were different from MSC isolated from good outcome recipients. Our results demonstrate that although lung-derived MSC show similarities with BM-derived MSC, they have a number of lung-specific properties. Furthermore, we demonstrate for the first time that MSC isolated from good outcome lung-transplantation recipients are very similar to MSC isolated from patients suffering from chronic rejection, BOS.

## Results

### Morphology, CFU-f capacity and proliferation

**Figure 1:**
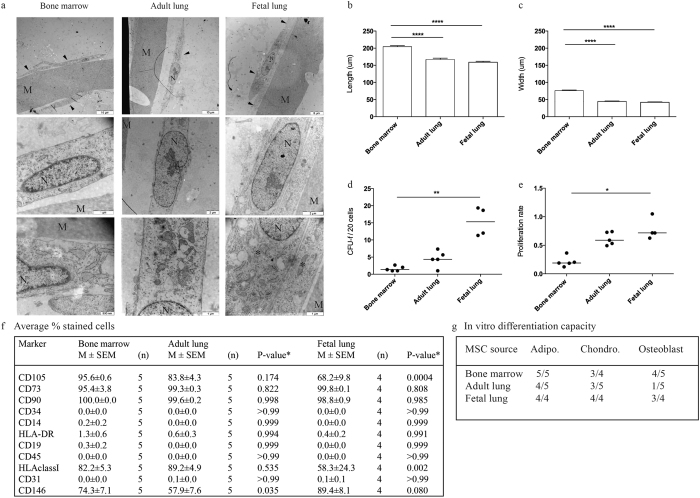
Electron microscopy pictures of MSC isolated from bone marrow, adult lung biopsies of lung-transplanted patients with good outcome and fetal lung tissue. M = membrane of the insert, N = nucleus, arrowhead = MSC and *  = mitochondria (**a**). Bone marrow derived MSC are longer (**b**) and wider (**c**) compared to lung derived MSC. Data are presented as mean (±SEM) and statistical analysis was performed by the Kruskal-Wallis test combined with Dunn’s multiple comparisons test, ***p < 0.001. Fetal lung derived MSC display a higher frequency of colonies compared to bone marrow MSC and adult lung MSC (**d**). Furthermore, fetal lung MSC have a higher proliferation rate compared to adult lung and bone marrow derived MSC (**e**). Data are shown as absorbance after 48 h (measured at 595 nm) subtracted by the 6 h absorbance. Data are presented as median and statistical analysis was performed by the Kruskal-Wallis test combined with Dunn’s multiple comparisons test, **p < 0.01, *p =  < 0.05. The surface marker profiles are presented as percent positively stained cells. Data are presented as mean (±SEM) and statistical analysis was performed by 2way ANOVA combined with Tukey’s multiple comparisons test (**f**). The *in vitro* differentiation capacity (adipocyte, chondrocyte and osteoblast) of MSC isolated from bone marrow, adult lung and fetal lung tissue. Data are presented as number of cultures that were able to differentiate per total number of tested cultures (**g**).

Cultured MSC isolated from adult lung biopsies of lung-transplanted patients with good outcome and fetal lung displayed similar morphological properties, but differed from BM-derived MSC (Fig. 1a). BM-derived MSC were significantly larger compared to lung-derived MSC both in width (p < 0.0001 and p < 0.0001, ANOVA <0.0001) and length (p < 0.0001 and p < 0.0001, ANOVA <0.0001) (Fig. 1b,c). Fetal MSC demonstrated a higher CFU-f (colony-forming unit, fibroblast) capacity compared to BM (p = 0.0055) and adult lung-MSC (p = 0.1728) (ANOVA = 0.0007), whereas no significant differences were seen between the MSC isolated from BM and adult lung tissue (p = 0.5890) (Fig. 1d). BM-derived MSC had a lower proliferation rate compared to adult and fetal lung MSC (p = 0.0574 and p = 0.0131 respectively, ANOVA = 0.0013), whereas no significant differences were seen between fetal and adult lung MSC (Fig. 1e).

### Surface marker profile and differentiation capacity

Cultured MSC were stained with a panel of monoclonal antibodies for surface marker expression analysis by flow cytometry. MSC from all sources expressed CD73, CD90, CD105, CD146 and HLA class I, but did not express the markers CD14, CD19, CD31, CD34, CD45 and HLA-DR (Fig. 1f). Lung-derived MSC displayed a similar surface marker profile compared to BM-derived MSC, with the exception of CD105 and HLA class I that were significantly higher expressed on BM-derived MSC compared to fetal lung MSC (p = 0.0004 and p = 0.002, respectively) and CD146 that was significantly higher expressed on BM-MSC compared to adult lung-MSC (p = 0.0352) (Fig. 1f). The surface marker expression between adult and fetal lung MSC was very similar with the exception of HLA class I and CD146 (p < 0.0001 and p =  < 0.0001, respectively). MSC from all cell sources could be differentiated into adipocytes and chondrocytes, whereas osteoblastic differentiation was impaired in adult lung MSC compared to BM and fetal lung cells (Fig. 1g, Supplementary figure 1).

### Cytokine expression

**Figure 2:**
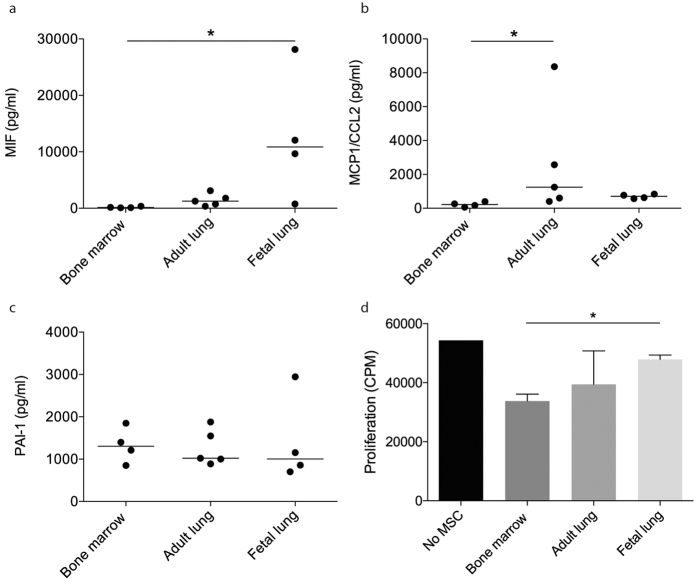
The production of Macrophage migration inhibitor factor (MIF) (**a**) Monocyte chemotactic protein 1/CCL2 (**b**) and Plasminogen activator inhibitor-1/Serpin E1 (**c**) was measured in conditioned medium (24 hours of culture). Data are presented as median and statistical analysis was performed by the Kruskal-Wallis test combined with Dunn’s multiple comparisons test. *p < 0.05. The lymphocyte suppressing effect of MSC were analyzed by Phytohemagglutinin (PHA) stimulation of lymphocytes (**d**). Data are presented as mean (±SEM) and statistical analysis was performed by using the student’s t-test. *p < 0.05.

Conditioned medium (24 hours of culture), isolated from BM-derived MSC, adult and fetal lung tissue, was screened for 36 cytokines using a human cytokine array panel (Supplementary figure 2). Based on the screening results, additional analyses using ELISAs were performed to exactly quantify the levels of Macrophage migration inhibitor factor (MIF), Monocyte chemotactic protein 1/CCL2 (MCP-1) and Plasminogen activator inhibitor-1/Serpin E1 (PAI-1). Fetal lung MSC produced higher concentrations of MIF compare to BM (p = 0.0111, ANOVA = 0.0035) and adult lung MSC (p = 0.5994), whereas no significant differences were found between BM and adult lung MSC (p = 0.2253) (Fig. 2a). BM-derived MSC secreted lower amounts of MCP-1/CCL2 compared to adult lung MSC (p = 0.0197) and fetal lung MSC (p = 0.1104) (ANOVA = 0.0068), but no differences were found between fetal and adult cells (p > 0.9999)(Fig. 2b). When measuring the expression of Serpin E1/PAI-1 secreted into the medium no significant differences were found between the different MSC sources (ANOVA = 0.8491) (Fig. 2c). The amount MIF, MCP-1, and PAI-1 in the control medium was below the detection limit.

### Effects of MSC on lymphocyte functions *in vitro*

The suppressive effects of MSC on lymphocytes were evaluated by adding 10% MSC to phytohemagglutinin (PHA) stimulated lymphocytes. There was no significant difference (p = 0.672) between the suppressive effects of BM-MSC (38% reduction, range: 30–44%) compared to adult lung MSC (27% reduction, range: −10–62%) on PHA stimulated lymphocyte proliferation. However, BM-MSC had a significantly higher (p = 0.010) suppressive effect compared to fetal lung MSC (12% reduction, range: 7–16%) (Fig. 2d).

### Gene expression

**Figure 3:**
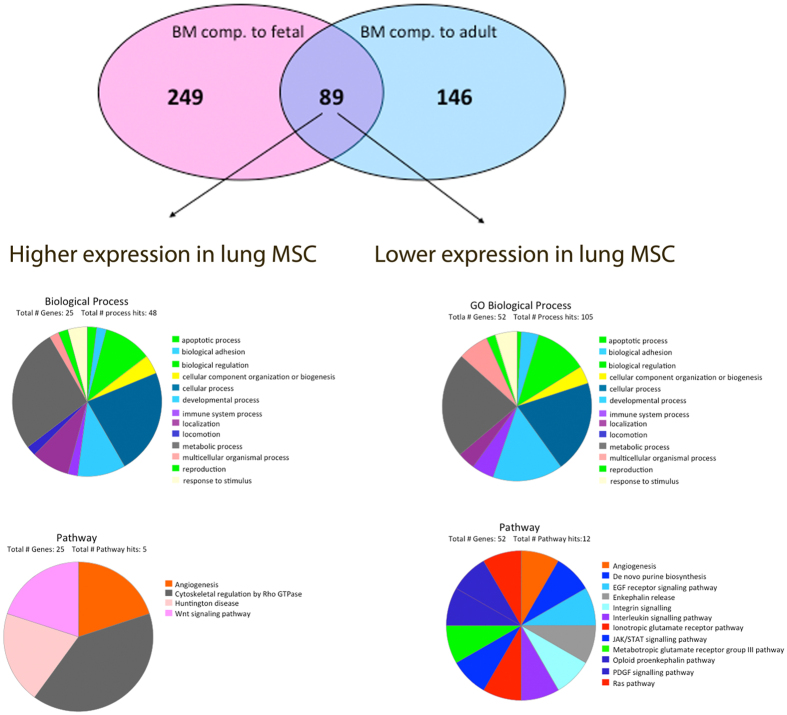
Total RNA extracted from MSC isolated from BM (n = 3), fetal (n = 3), and adult lung biopsies of lung-transplanted patients with good outcome (n = 3) was analyzed by microarray chip. We found 338 genes that were differentially expressed between bone marrow derived MSC and fetal lung MSC. When comparing bone marrow derived MSC to adult lung MSC 235 genes were found to be significantly differently expressed. Furthermore, 89 genes were found to be differently expressed when comparing lung derived MSC (both fetal and adult lung) with bone marrow derived MSC. Data are presented as a Venn diagram. Further, the 89 identified genes were divided into higher and lower expressed genes in lung MSC compared to BM-derived MSC and genes were further categorized into GO biological processes and GO pathways. The PANTHER classification system was used to further classify identified genes.

**Figure 4:**
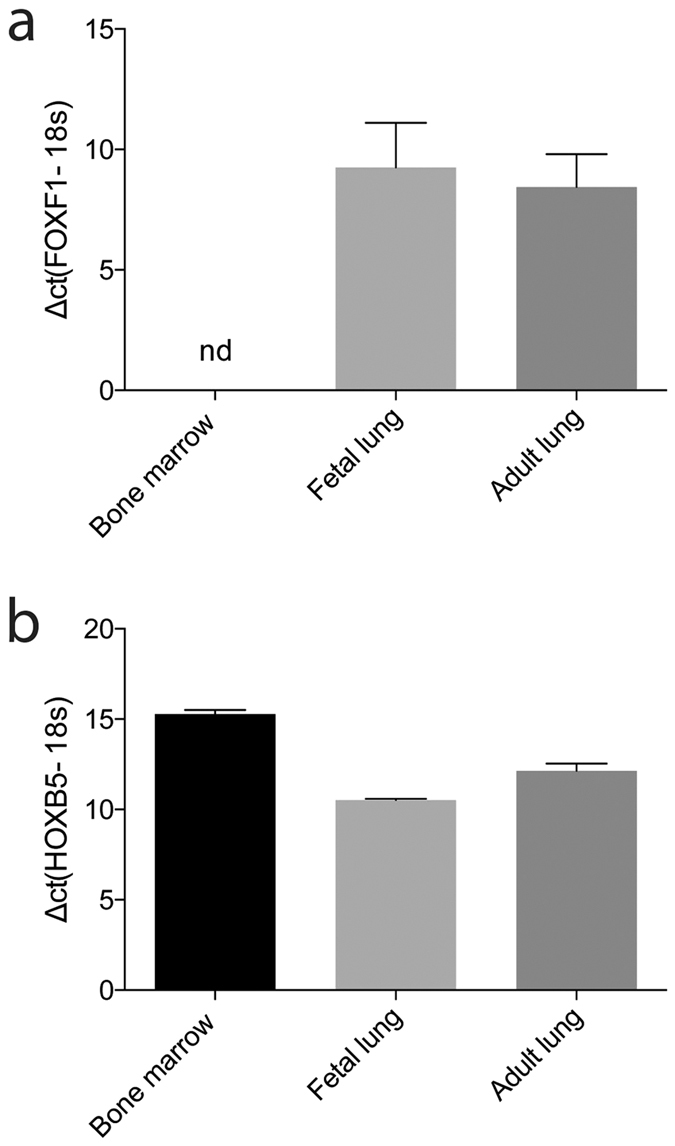
The gene expression of FOXF1 (**a**) and HOXB5 (**b**) was validated by q-PCR analysis on total RNA isolated from bone marrow derived MSC (n = 3), MSC isolated from adult lung biopsies of lung-transplanted patients with good outcome (n = 3) and fetal lung derived MSC (n = 3). Data are presented as median and range. Nd = not detectable.

In order to examine differences in gene expression profile, total RNA of MSC isolated from BM, fetal and adult lung was analyzed using a standard microarray chip. The gene expression profiles of adult lung-derived and fetal lung-derived MSC were very similar compared to BM-derived MSC, with only 235 and 338 genes, respectively, being significantly differently expressed. Out of the 235 genes that were significantly different between adult lung-derived and BM-derived MSC, 90 genes were higher expressed in the lung-derived MSC. In case of fetal lung-derived MSC, 166 genes were higher expressed compared to the BM-derived MSC. Interestingly, only 89 genes were found to be expressed differently when comparing BM-derived MSC to both, fetal- and adult lung-derived MSC, indicating that these genes are lung-specific (Fig. 3). Most of these genes were annotated to cellular processes and metabolic functions, but also genes related to locomotion, immune system processes and response to stimulus were identified. Among the identified genes, we found genes involved in epithelial-mesenchymal transition (EMT) such as SNAI2, HGF, TGF-beta 2 and 3, TCF4, and CDH2. Genes involved in hematopoietic support, such as VCAM 1, CXCL12 and EGR1 was also found among the identified genes, and VCAM1 was found to be significantly higher in BM-MSC compared to lung-derived MSC (Supplementary table 3). Furthermore, secreted frizzled-related protein 1 (SFRP1), a gene involved in the Wnt-signaling pathway was found to be higher expressed in adult and fetal lung-derived MSC compared to BM-derived MSC (29.5 and 20.4 fold change, respectively). In addition, typical lung-related genes such as FOXF1 and HOXB5 were found to be higher expressed by adult and fetal lung-derived MSC compared to BM-derived MSC (FOXF1: 13.1 and 13.6 fold change, respectively and HOXB5: 12.6 and 13.1 fold change, respectively) (Fig. 3, Supplementary table 1). FOXF1 and HOXB5 expression in lung-derived MSC was furthermore confirmed by q-PCR (Fig. 4). BM-derived MSC on the other hand, expressed lower amounts of HOXB5 and showed no expression of FOXF1 (Fig. 4). In addition, the microarray analysis showed that adult and fetal lung-derived MSC were very similar with only 65 genes being significantly differently expressed in these tissues (Supplementary table 2). Among these 65 genes, higher expression in fetal lung MSC compared to adult lung MSC was observed for genes related to developmental processes such as Tubulin beta-2B chain (TUBB2B) and Fibrillin-2 (5.2 and 8.9 fold change, respectively). Furthermore, we found a lower expression of immune system process-related genes such as glutathione peroxidase 3 and major histocompatibility complex class I-related gene protein (MR1) in fetal lung MSC compared to adult lung MSC (6.4 and 3.1 fold change, respectively) (Supplementary table 2).

### MSC isolated from lung-transplanted patients with BOS are very similar to good outcome recipients

**Figure 5:**
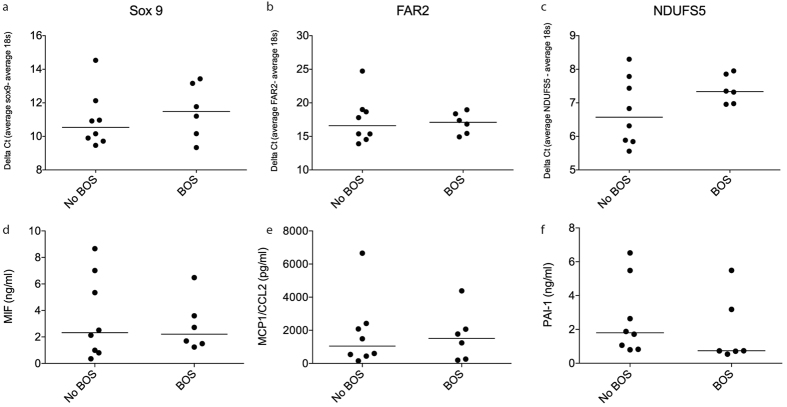
MSC isolated from lung-transplanted patients with a chronic rejection (n = 6) showed no difference in the gene expression of Sox 9 (**a**) FAR2 (**b**) and NDUFS5 (**c**) compared to the good outcome recipients (n = 8) when analyzed total RNA by q-PCR. Data are presented as median and statistical analysis was performed by non-parametric Mann-Whitney test. The production of Macrophage migration inhibitor factor (MIF) (**d**) Monocyte chemotactic protein 1/CCL2 (**e**) and Plasminogen activator inhibitor-1/Serpin E1 (**f**) was measured in conditioned medium (24 hours of culture) from adult lung MSC with and without the development of BOS. Data are presented as median and statistical analysis was performed by the non-parametric Mann-Whitney test. *p < 0.05.

Next, we went on to evaluate if MSC isolated from biopsies of lung-transplanted patients with BOS differed compared to patients without BOS, *i.e.* good outcome recipients. Here we found that Sox9, FAR2, LOC728855, and NDUFS5 were significantly higher expressed in MSC isolated from BOS patients (Supplementary figure 3). However, q-PCR analysis showed no differences in the expression of Sox 9 (p = 0.4908), FAR2 (p = 0.9497) and NDUFS5 (p = 0.2284) in lung-transplanted patients with chronic rejection (n = 6) compared to the good outcome recipients (n = 8) (Fig. 5a–c). No differences in cytokine production were found in BOS MSC compared to good outcome patients (Fig. 5d–f). Furthermore, no significant differences were observed for proliferation rates (Supplementary figure 3d), CFU-f potential (Supplementary figure 3c), surface marker profile (Supplementary figure 3e) and differentiation potential (Supplementary figure 1). The only differences between the two MSC groups that could be detected were related to morphology, *i.e*. BOS MSC had a decreased length (p = 0.0048) and an increased width (p = 0.0004) (Supplementary figure 3a,b).

## Discussion

In spite of the fact that MSC are used as cell-based therapy for various severe lung diseases, there is very limited knowledge regarding primary lung tissue-derived MSC in health and disease. Furthermore, differences and similarities between BM-derived MSC, which are often used in the clinical trials, and tissue-resident lung MSC have not been thoroughly investigated yet. Therefore, the current study aimed to compare tissue-resident lung-derived MSC to the well-characterized BM-derived MSC in order to evaluate whether or not lung-derived MSC possess tissue-specific properties. Furthermore, we aimed to evaluate if MSC isolated from lung-transplanted patients with BOS are different compared to patients without BOS, *i.e.* good outcome recipients.

Our results demonstrate that BM-derived MSC are larger, have a lower proliferation rate and a lower CFU-F potential compared to lung-derived MSC. Interestingly, and in accordance to our previously published data are lung-derived MSC characterized by an impaired osteogenic differentiation potential^5^. Theoretically, this might be an advantage when developing cell therapies for solid organs like the lung where ossification should be avoided. In fact, calcification and bone formation, although not normally occurring in lung tissues, have been described in very rare conditions, such as pulmonary alveolar microlithiasis (PAM), diffuse pulmonary ossification (DPO) and ossification in carcinoid tumors^6^,^7^,^8^. Adult lung MSC secreted significantly higher amounts of the cytokine MCP-1, which is known to be important for innate immunity by attracting monocytes. This could indicate that tissue-resident MSC worsen an inflammatory reaction and, accordingly increased amounts of MCP-1 have been found in patients with BOS^9^. However, MCP-1 has also been demonstrated to play an important role in the protection against gram-positive and gram-negative bacteria^10^.

To further investigate possible differences between lung- and BM-derived MSC, we investigated possible gene expression differences utilizing standard microarrays. This analysis revealed that lung-derived and BM-derived MSC have very similar gene expression profile. However, 89 genes were found to be significantly different when comparing lung-derived MSC (fetal and adult) with BM-derived MSC, *i.e.* these genes were lung-specific. Accordingly, genes such as HOXB5 and FOXF1, which have been demonstrated to be very important for human lung development and branching^11^, were found to be higher expressed in lung-derived MSC compared to BM-derived MSC. Furthermore, secreted frizzled-related protein 1 (SFRP1) was found to be higher expressed by lung-derived MSC compared to BM-derived MSC. SFRP1 is a protein involved in the Wnt-signaling acting as an antagonist by binding to Wnt-proteins and thereby preventing signal activation^12^. A study published by Salazar *et al*. demonstrated that SFRP1 inhibited the proliferation of primary human lung fibroblasts^13^. Furthermore, Shiomi *et al*. have demonstrated that SFRP1 played an important role in lung development, but also in tissue repair by maintaining progenitor cells in their undifferentiated state^14^,^15^.

Fetal- and adult lung-derived MSC share many similarities *e.g.* morphology, proliferation rate and cytokine secretion (MCP-1 and PAI-1/Serpin E1). Fetal lung-derived MSC display a high expression of MIF, which has been reported to play a role in lung maturation^16^. Surface marker expression of fetal- and adult lung-derived MSC was comparable, except for the expression of CD146 that was significantly increased in adult lung MSC, and HLA class I, which was significantly decreased in adult lung MSC. An increased expression of HLA class I in adult MSC compared to fetal MSC has previously been demonstrated by Gotherstrom *et al*.^17^. Last, the microarray analysis demonstrates that adult and fetal lung-derived MSC have similar gene expression profile, which only 65 genes being differentially expressed between the two cell types. The glutathione peroxidase 3 gene, which function is to protect cells from oxidative damage generated by oxidizing glutathione and hydrogen peroxide, was found to be higher expressed in adult lung-derived MSC. Interestingly, down-regulation of glutathione peroxidase 3 has been associated with the progression of lung cancer^18^.

One important aim of the current study was to compare MSC isolated from lung tissues of lung-transplanted patients with BOS and good outcome recipients. To the best of our knowledge this is the first study address this question and our data indicate that MSC from BOS patients are very similar to MSC from good outcome recipients. No significant differences were found between the two groups in gene expression profile and cytokine release. Furthermore, no differences in surface marker profile were found between MSC isolated from BOS patients compared to good outcome recipients.

Taken together, this study demonstrates that tissue-resident lung-derived MSC share many similarities with the well-characterized BM-derived MSC. However, they also possess unique lung-specific properties, which might be important for future studies aiming to investigate the functional role of tissue-resident lung MSC in health and disease. Furthermore, we report for the first time that MSC isolated from lung biopsies obtained from lung-transplanted patients with BOS have very similar properties as MSC isolated from good outcome recipients, indicating that the phenotype of lung tissue MSC are not altered in BOS patients.

## Methods and Materials

### Cell isolation

#### Lung-derived cells

Adult lung-derived MSC were isolated from peripheral transbronchial (parenchymal tissue) biopsies obtained from lung-transplanted patients as previously described^5^. Briefly, transbronchial biopsies were collected, washed and cells were isolated by enzymatically digestion (collagenase, hyaluronidase and DNase). After digestion, the cells were washed and seeded into StemMACS MSC Expansion medium (MACS Miltenyi Biotec)^5^. Patients were divided into two groups, *i.e.* patients developing BOS (n = 8, average age: 55.4 years) and good outcome recipients without BOS (n = 6, average age: 49.2 years), according to the International Society for Heart and Lung Transplantation guidelines (Supplementary table 4)^19^. Good outcome recipients were defined to have lung function parameters above 80% of baseline as measured by standard spirometry^19^. This study was approved by the Swedish regional (Lund) ethical committee (application number: 2005-560), and all patients gave their written informed consent to participate in the study. All experimental protocols were carried out in accordance with approved guidelines from the Swedish regional (Lund) ethical committee (application number: 2005-560). Fetal lung cells (n = 4) with an estimated developmental age of 16–17.5 weeks were purchased from Novogenix Laboratories LLC, LA, USA (Supplementary table 4). MSC were grown in StemMACS MSC Expansion medium (MACS Miltenyi Biotec) after adherence on standard plastic tissue flasks. In the CFU-F assays, primary cells were seeded at clonal density, whereas MSC expansion cultures were not performed at clonal density. Medium was changed weekly and cells were passaged with 0.05% trypsin-EDTA at 70–90% confluence. One of the fetal cell samples was FACS sorted and the fraction of CD90+/EpCAM−/CD31- before purchased. Cultured MSC isolated from adult- and fetal lung tissue were used at passage 2–4 in all experiments.

#### Bone marrow cells

Bone marrow was aspirated (n = 5; average age: 26.8 years) from the iliac crest bone of consenting healthy donors as previously described (Supplementary table 4)^20^. This study was approved by the Swedish regional (Lund) ethical committee, and all methods were carried out in accordance with approved guidelines (application number: 2009/532). Bone marrow mononuclear cells (MNC) were isolated by density-graded centrifugation and cells were further seeded in StemMACS MSC Expansion medium. BM-MNC were seeded at clonal density in the CFU-F assays, whereas expansion of BM-derived MSC were not performed at clonal density. Medium was changed weekly and cells were passaged with 0.05% trypsin-EDTA at 70–90% confluence. BM-MSC in passage 2–4 were used in all experiments.

### MSC Characterization

#### Morphology (Crystal violet, Transmission electron microscopy)

Cultured MSC were harvested by 0.05% Trypsin-EDTA and cells were seeded in four-well chamber slides (10.000 cells/well) and allowed to adhered over night. After attachment cells were fixed with 1% glutaraldehyde and stained with crystal violet dye. Pictures were taken and the length and width was recorded for 100 cells per sample using the Visiopharm^TM^ software (Visiopharm, Hoersholm, Denmark). For visualization, 2 × 10^4^ MSC were seeded into trans well inserts (cat no. 3422, Corning, NY, USA) and was left to adhere over night. Cells were fixed with 1.5% paraformaldehyde and 1.5% glutaraldehyde in 0.1 M Sorensen phosphate buffer for one hour, washed and dehydrated with increasing concentration of acetone. The specimen was embedded in Polybed epoxy resin. The specimen block was sectioned on a Leica EM UC7 ultramicrotome and stained with 4% uranyl acetate and 1% lead citrate. The section was analyzed and photographed using a FEI Tecnai Spirit Bio TWIN transmission electron microscope.

#### CFU-F (Colony forming unit-fibroblast)

The frequency of CFU-F in lung and bone marrow cells were examined as described previously^20^,^21^. Briefly, cultured MSCs were plated in six-well plates at 10, 20, 50 and 100 cells/well. Medium was completely changed after 3 and 7 days. On day 14, cells were fixed and stained with crystal violet. CFU-Fs were enumerated as colonies with ≥40 fibroblast-like cells. Assays were set up in triplicates.

#### Proliferation

The proliferation rate of lung-derived and BM-derived MSC was evaluated as previously described^22^. Briefly, cells were seeded in 96-wells and fixed after 6, 25, 48 and 73 hours. Cells were stained with Crystal Violet dye and absorbance was measured at 595 nm on a spectrophotometer plate reader. Assays were set up in five replicates.

#### Flow cytometry

Cultured cells were harvest, non specific binding was blocked and the cells were stained with different combinations of the following directly-conjugated antibodies: CD105 (cat no. 561443, BD Pharmingen), CD73 (cat no. 550257, BD Pharmingen), CD90 (cat no. 559869, BD Pharmingen), CD34 (cat no. 555821, BD Pharmingen), CD14 (cat no. 345785, BD), CD19 (cat no. 555415, BD Pharmingen), CD31 (cat no. 555445, BD Pharmingen), CD45 (cat no. 345808, BD), CD146 (cat no. 550315, BD Pharmingen), HLA-DR (cat no. 555558, BD Pharmingen) and HLA class I (cat no. 555553, BD Pharmingen) as described before^5^. 7-amino-actinomycin D (Sigma Aldrich) was used for dead cell exclusion. Samples were analyzed on a FACSCalibur (BD Bioscience) and data acquisition and analysis were performed using CellQuest (BD Bioscience) and FlowJo software (Tree star, Ashland, Oregon, USA) respectively. Results are presented as percent positively stained cells.

#### *In vitro* differentiation

The *in vitro* differentiation potential of cultured MSC was evaluated as previously described^5^,^20^. Briefly, cells were cultured in osteogenic induction medium (Dulbecco’s modified Eagle’s medium high glucose/L-glutamine (PPA) containing β-glycerophosphate (Sigma Aldrich), L-ascorbic acid-2-phosphate (Wako Chemicals, Neuss, Germany) and dexamethasone (Sigma Aldrich) followed by a Alizarin Red (Sigma Aldrich) staining. For adipocyte differentiation, cells were seeded in AdipoDiff medium (Miltenyi Biotec) and further stained with Oil Red O (Sigma Aldrich). Pictures of adipocyte and osteoblast differentiation were taken with a Nikon Eclipse TE2000-E microscope equipped with a Nikon DS-U2/L2 USB camera using NIS Elements software. Chondrogenic differentiation was performed by culturing cell pellets in chondrocyte induction medium (Miltenyi Biotec) supplemented with TGF-β. Chondrocyte pellets were fixed and frozen in O.C.T (Tissue-Tek, Sakura, Zoeterwoude, The Netherlands). The pellets were sectioned and stained against Aggrecan (cat no. AF1220, R&D Systems). Pictures of chondrocyte pellets were taken with a Nikon TE200E microscope equipped with Olympus DP80 camera using CellSense software.

#### Lymphocyte proliferation assay

Mitogen stimulation assays were performed as described previously^23^. Briefly, blood samples were taken from consenting healthy donors, and mononuclear cells were isolated by standard ficoll separation (Lymphoprep^TM^, Axis Shield PoC AS, Norway). 1 × 10^5^ cells from a single donor (incubated with (^3^H) thymidine) were stimulated with phytohemagglutinin (PHA, 20 ug/ml) and co-cultured with 10% irradiated MSC (20 Gy) in RPMI 1640 (Gibco BRL) supplied with 1% L-glutamine (Gibco), 1% PeSt (Gibco) and 10% human, heat inactivated AB serum (pooled). After 4 days of culture, cell proliferation was assessed by (^3^H) thymidine incorporation. Assays were setup in triplicates.

#### Cytokine analysis

Conditioned medium from BM-MSC (n = 2), adult lung MSC (n = 2) and fetal lung MSC (n = 2) were collected after 24 hours of culture and stored at −20 °C. The conditioned medium was screened for 36 different cytokines using the Human Cytokine Array Panel A antibody array (cat no. ARY005, R&D Systems, Minneapolis, USA) according to manufacturer’s instructions. The arrays were scanned and pixel density measurements were obtained with Quantity One software (Bio-Rad). Based on the cytokine array results, ELISA was performed for the cytokines MIF (cat. No. DMF00B), SerpinE1/PAI-1 (cat. no. DSE100) and MCP-1/CCL2 (cat. no. DCP00), all from R&D Systems. Conditioned medium from BM (n = 4), good outcome recipients/adult lung without BOS (n = 8), BOS patients (n = 6) and fetal lung (n = 4) were analyzed according to manufactory’s instructions. DMEM without serum were used for MIF and Serpin E1/PAI-1 analysis and for MCP-1/CCL2 ELISA we used DMEM containing 2% FCS. The cytokine measurements of BM, good recipients/adult lung (no BOS) and fetal lung derived MSC were normalized to proliferation rate. The cytokine measurements of MSC derived from adult lung with BOS and good outcomes recipients were not normalized to proliferation, because they displayed similar proliferation rates.

#### Microarray/q-PCR

Total RNA was extracted from BM-MSC (n = 3), adult lung MSC without BOS (n = 3), adult lung MSC with BOS (n = 3), and fetal lung MSC (n = 3) with RNeasy mini kit (Qiagen, GmBH, Hilden, Germany) according to the manufacturer’s instructions. The RNA amount and quality was measured using NanoDrop ND-100 (Nano Drop Technologies, Delaware, Maryland, USA) and for microarray the RNA integrity was evaluated by Agilent 2100 Bioanalyzer (Agilent Technologies, CA, USA). For the microarray analysis the RNA was analyzed with basic HumanHT-12 Illumina chip and Experimental Quality Analyses were performed using GenomeStudio software V2011.1. Probe summarization and data normalization was performed using robust multi-array analysis (RMA)^24^. Probe sets that did not have signal intensity above the median of negative control intensity signals in 80% of the samples were excluded. Furthermore, probe sets that did not have gene annotations or expired annotations were filtered out. Signals were log 2 transformed. Data was uploaded to the GEO (reference number: GSE74163). For q-PCR, total RNA (500 ng) was reverse-transcribed using QuantiTect reverse transcription kit (Qiagen) and stored at −80 °C. cDNA was mixed with QuantiTect Sybr green kit (cat. no. 204143, Qiagen). The samples were held at 95 °C for 15 min and then 40 cycles at 94 °C for 15 sec, 55 °C for 30 sec and then 72 °C for 30 sec. Reactions were performed using Stratagen MX3005P. The following primers assays were used: Hs_SOX9_1_SG QuantiTect Primer Assay (cat. no. QT00001498) 111 (NM_000346), Hs_FAR2_1_SG QuantiTect Primer Assay (cat. no. QT00043750), Hs_NDUFS5_1_SG QuantiTect Primer Assay (cat. no. QT00079079), Hs_RRN18S_1_SG QuantiTect Primer Assay (cat. no. QT00199367), Hs_HOXB5_1_SG QuantiTect Primer Assay (cat. no. QT00026740) and Hs_FOXF1_1SG QuantiTect Primer Assay (cat. no. QT00029687) (all from Qiagen). LOC728855 was not evaluated since this gene has an unknown function.

#### Statistical analysis

Morphology data, lymphocyte suppression data and surface markers are presented as mean (±SEM). CFU-F data, proliferation data, cytokine data and q-PCR data are presented as median. The non-parametric Mann-Whitney test was used to compare statistical differences between two groups. The non-parametric Kruskal-Wallis test combined with Dunn’s multiple comparisons test was used to compare statistical differences between three groups. The comparison between surface marker expressions of two and three groups was performed by 2wayANOVA combined with Bonferroni’s and Tukey’s multiple comparisons test, respectively. For lymphocyte suppression assays the parametric student’s t-test was used to compare statistical differences. Analyses were performed with GraphPad Prism software V.6.0 g. p-values ≤ 0.05 were considered as significant. For the microarray, SAM analysis using TMEV v4.0 software was performed to identify significantly differentially expressed genes between groups^25^. We used AmiGO database to map GO term for each gene symbol. R package was used to create Venn diagram. The PANTHER classification system (pantherdb.org) was used for categorizing the identified genes into biological process categories and GO pathways.

## Additional Information

**How to cite this article**: Enes, S. R. *et al*. MSC from fetal and adult lungs possess lung-specific properties compared to bone marrow-derived MSC. *Sci. Rep.* **6**, 29160; doi: 10.1038/srep29160 (2016).

## Supplementary Material

Supplementary Information
