# Supplementary material for: MSC from fetal and adult lungs possess lung-specific properties compared to bone marrow-derived MSC
Source: Sci Rep. 2016 Jul 6;6:29160. doi: 10.1038/srep29160 (PMC4933903; doi:10.1038/srep29160)
Supplement: Supplementary Information [file srep29160-s1.doc]

MSC from fetal and adult lungs possess lung-specific properties compared to bone marrow-derived MSC

Sara Rolandsson Enes, Annika Andersson Sjöland, Ingrid Skog, Lennart Hansson, Hillevi Larsson, Katarina Le Blanc, Leif Eriksson, Leif Bjermer, Stefan Scheding, Gunilla Westergren-Thorsson

Supplementary figure 1.

**
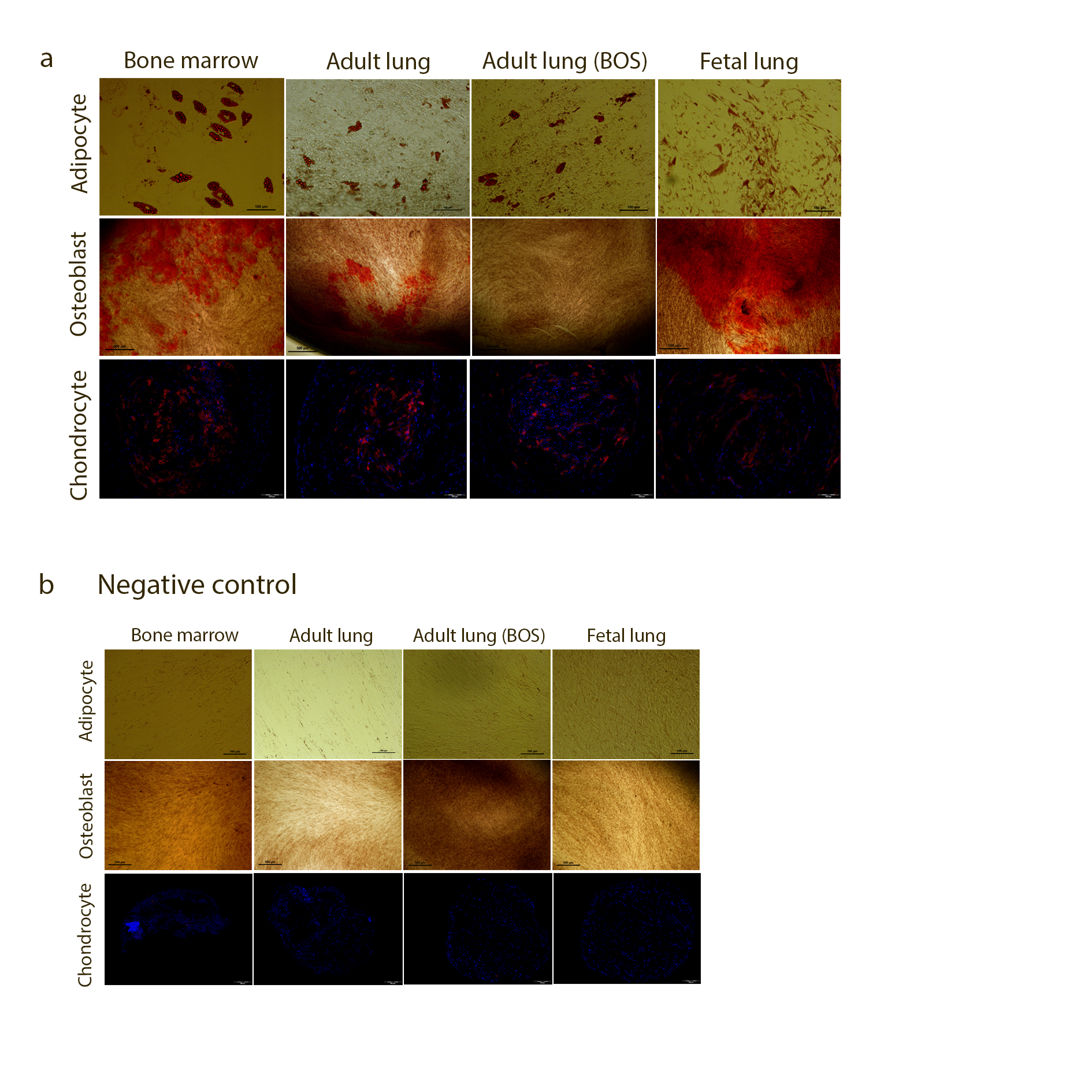
**Supplementary Figure 1. MSC isolated from bone marrow (n=4-5), fetal lung (n=4), adult lung without BOS (n=5) and adult lung with BOS (n=4) were cultured in adipocyte, osteoblast and chondrocyte induction medium (a). As negative controls, cells were cultured in normal growth medium (b). Scale bar for adipocyte differentiation: 100μm. Scale bar for osteoblast differentiation: 500μm. Scale bar for chondrocytes: 100 μm.

Supplementary Figure 2.

Supplementary Figure 2. Conditioned medium (24 hours of culture) harvested from cultures of BM-derived MSC (a, h), adult lung (no BOS) (b, i), adult lung with BOS (c, j) and fetal lung tissue MSC (d, k), were screened for 36 cytokines using the human cytokine array panel A. Macrophage migration inhibitor factor (MIF) (e), Monocyte chemotactic protein 1/CCL2 (MCP-1) (f) and Plasminogen activator inhibitor-1/Serpin E1 (PAI-1) (g) were chosen from the screening results for validation measurements using ELISA.

Supplementary Figure 3.

Supplementary Figure 3. Adult lung-derived MSC isolated from patients with BOS are shorter (a), but wider (b) compared to lung-derived MSC from good outcome recipients (no BOS). Data are presented as mean (± SEM) and statistical analysis was performed by non-parametric Mann-Whitney test, ** = p<0.01 *** = p<0.001. No significant differences were found in the capability of forming colonies (c) or proliferation rates (d) between the two groups. Individual data are presented with the median indicated as horizontal line. Surface marker expression profiles are presented as percent positive cells for lung-derived MSC from patients without (no BOS) and with BOS (e). Data are presented as mean (± SEM). A microarray analysis was performed on lung-derived MSC from patients without (no BOS) and with BOS (n=3/group). MSC derived from lung-transplanted patients with BOS showed an increased expression of four genes compared to good outcomes recipients (f)
